# Effects of FGFR2 kinase activation loop dynamics on catalytic activity

**DOI:** 10.1371/journal.pcbi.1005360

**Published:** 2017-02-02

**Authors:** Jerome M. Karp, Samuel Sparks, David Cowburn

**Affiliations:** 1 Department of Biochemistry, Albert Einstein College of Medicine, Bronx, New York, United States of America; 2 Department of Physiology and Biophysics, Albert Einstein College of Medicine, Bronx, New York, United States of America; CNAG - Centre Nacional d’Anàlisi Genòmica and CRG - Centre de Regulació Genòmica, SPAIN

## Abstract

The structural mechanisms by which receptor tyrosine kinases (RTKs) regulate catalytic activity are diverse and often based on subtle changes in conformational dynamics. The regulatory mechanism of one such RTK, fibroblast growth factor receptor 2 (FGFR2) kinase, is still unknown, as the numerous crystal structures of the unphosphorylated and phosphorylated forms of the kinase domains show no apparent structural change that could explain how phosphorylation could enable catalytic activity. In this study, we use several enhanced sampling molecular dynamics (MD) methods to elucidate the structural changes to the kinase’s activation loop that occur upon phosphorylation. We show that phosphorylation favors inward motion of Arg664, while simultaneously favoring outward motion of Leu665 and Pro666. The latter structural change enables the substrate to bind leading to its resultant phosphorylation. Inward motion of Arg664 allows it to interact with the **γ**-phosphate of ATP as well as the substrate tyrosine. We show that this stabilizes the tyrosine and primes it for the catalytic phosphotransfer, and it may lower the activation barrier of the phosphotransfer reaction. Our work demonstrates the value of including dynamic information gleaned from computer simulation in deciphering RTK regulatory function.

## Author summary

Receptor tyrosine kinases are proteins integral to relaying signals from outside the cell to activators inside the cell that stimulate cell growth and development. Therefore, when these proteins show intrinsic activity independent of extracellular signaling, they can frequently cause developmental abnormalities, if the unchecked activity occurs before birth, or cancer, if the unchecked activity occurs later in life. Understanding what causes these proteins to become active upon receiving an extracellular signal will be helpful in pinpointing how they can exhibit activity without the extracellular signal. To study this phenomenon, we examined one receptor tyrosine kinase, FGFR2 kinase, and used computer simulation to identify what conformational changes occur in this protein upon activation. We then identified the function of these conformational changes in enabling the enzyme’s catalytic reaction to occur. Our results demonstrate the value of incorporating simulation data in analyzing the mechanisms of receptor tyrosine kinase activation, and suggest important features of this enzyme that should be considered in future drug development.

## Introduction

Receptor tyrosine kinases (RTKs) occupy a central role in cellular regulation, acting as intermediaries in relaying signals from extracellular ligands to major signaling pathways in the cell [1–3]. Although the structural elements of RTKs are well-conserved [4], their functions are widely divergent. This is due to the subtle differences in the sequences and dynamic properties of structural elements underlying kinase activity [5]. The similarities between the various RTKs combined with their divergent behaviors presents a unique challenge in designing drugs to target specific RTKs whose constitutive activity has pathologic consequences, without generating off-target effects caused by reduced activity of other kinases [6, 7]. This endeavor has had profound successes [8] but still requires additional effort, particularly with regard to filling the gaps in our structural knowledge of these proteins.

RTKs, like all kinases, have an N-lobe and C-lobe, with the active site generally in the pocket buried between them [4, 9]. In order to avoid pathologic constitutive activity, RTKs have several autoinhibitory mechanisms in place that prevent the substrate from accessing the active site or prevent the phosphotransfer from taking place [10–13]. Some of these regulatory mechanisms involve the extracellular, transmembrane or juxtamembrane domains of the kinase preventing association of two kinase domains and their resultant autophosphorylation. Other mechanisms are contained within the kinase domain itself and involve regulatory regions whose dynamics may either favor or disfavor catalytic activity. One regulatory region is the nucleotide-binding loop, often referred to as the P-loop, at the tip of the N-lobe near the active site, that binds the ATP molecule that donates a phosphate group to the substrate [4, 9]. A second regulatory region is the αC helix that makes contact with the activation loop and often undergoes large movements to form the catalytically active state of the kinase. A third regulatory region, which is usually post-translationally modified to alter its regulatory behavior, is the activation loop. The activation loop usually contains one or multiple tyrosine residues that are available to be phosphorylated by other enzymes or, in many cases, autophosphorylated. This phosphorylation leads to altered dynamics of the activation loop residues resulting in greater catalytic activity of the kinase [14–17].

The fibroblast growth factor receptors (FGFRs) are a superfamily of RTKs that activate the MAP kinase and PI3 kinase pathways [18, 19]. Binding of an activator of the fibroblast growth factor family in concert with heparan sulfate stabilizes the dimerization of two receptors’ extracellular domains, leading in turn to the apposition of the receptors’ intracellular kinase domains. As in other RTKs, the kinase domain contains an activation loop with two adjacent tyrosine residues. Apposition of the kinase domains enables the activation loops to undergo *trans*-autophosphorylation, rendering the kinases catalytically active and able to perform phosphorylation of tyrosine residues in FGFR kinase substrates including PLC-γ [20–22] and additional sites on FGFR kinases [23–25]. In this work, we focus on the FGFR2 kinase, for which a wealth of experimental structural information is available, including crystal structures of the wild type kinase [25, 26], of mutant kinases [15, 26, 27], and NMR chemical shift data [15]. Previous work suggests that the FGFR2 kinase activation loop toggles between two states, inactive and active, and that mutation of activation loop residues can perturb the balance between these two states to increase the time that the kinase is in the activated state, even without phosphorylation [15]. Crystal structures illustrate several structural changes that occur when FGFR2 kinase is activated. These include rearrangement of the activation loop, a small rotation of the N-lobe toward the C-lobe, and dissolution of a network of hydrogen bonds between side chains in a triad of residues known as the “molecular brake” [26, 28]. Genomic point mutations in the activation loop, the αC helix, or the molecular brake *in utero* frequently lead to developmental disorders [26, 29–31], while somatic mutations may lead to cancer [29, 30, 32]. Surprisingly, in contrast to most RTKs, there is little apparent motion of the αC helix in the FGFR kinases upon activation, with crystal structures showing that the helix moves together with the rest of the N-lobe. This suggests that the bulk of structural change in the activated kinase is concentrated in the activation loop structure. Thus it is especially crucial to investigate the details of activation loop rearrangement in order to understand FGFR2 kinase function.

Despite the many crystal structures of FGFR2 [33], a mechanism to explain how phosphorylation of the activation loop residues leads to catalytic activity has not yet surfaced. In FGFR1, the activation loop residues Arg661 and Pro663 block the active site in the inactive structure, but in the active structure change conformation to allow a substrate to bind [11]. This led to the hypothesis that phosphorylation of the activation loop alters its structure to move these two residues away from the active site, allowing substrate phosphorylation. However, neither the inactive nor the active crystal structure of FGFR2 (PDBs 2PSQ and 2PVF, respectively [26]) shows any activation loop residues in the active site. In this study, we use molecular dynamics (MD) simulation to probe the dynamics of FGFR2 kinase and propose a mechanism to explain the regulatory role of activation loop phosphorylation.

## Results

### Backbone and sidechain motions in activation loop reorganization

In order to visualize the process by which the inactive structure of the activation loop undergoes conformational transition(s) into the active structure, we used the string method in collective variables [34]. The string method finds the minimum free energy path (MFEP) connecting two states at the end points, in this case the inactive and active structures of the kinase. The MFEP is the most likely pathway that the system will use to transition from the inactive structure to the active structure [35]. As collective variables, we used the Cα atoms of the activation loop residues and the αC helix, as well as important activation loop side chain atoms, as described in Methods. In addition, we included the Nδ2 atom of Asn549 and the Cδ atom of Glu565, as these two atoms are part of the network of hydrogen bonds termed the “molecular brake,” which has been proposed to play a regulatory role in FGFR2 kinase activation [26].

**Fig 1:**
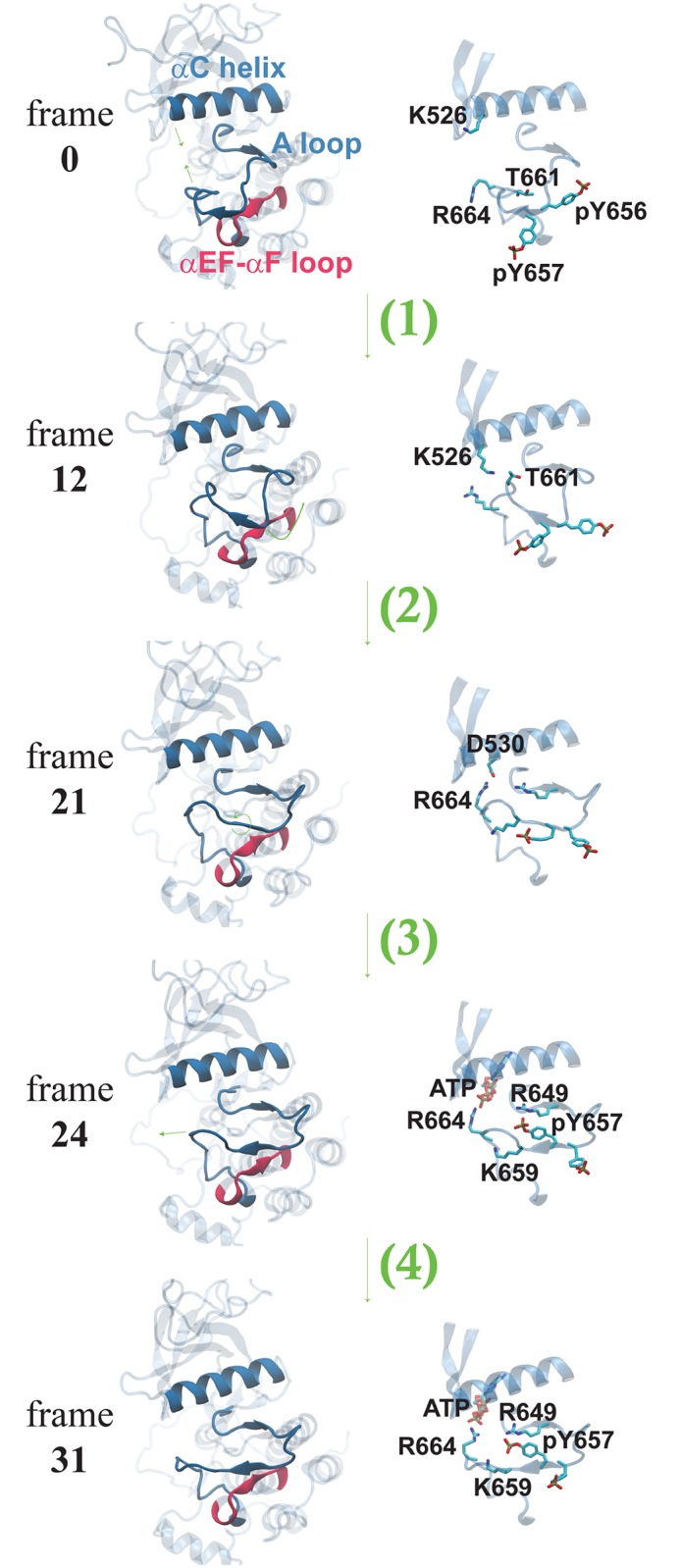
Minimum free energy path (MFEP) determined by the string method in collective variables. The left column shows four changes to the backbone that occur in the pathway: (1) motion of residues 660–663 and αC helix toward one another; (2) sliding of αEF-αF loop along activation loop; (3) rotation of the backbone of the activation loop to form a β-strand; (4) outward motion of residues 660 through 663. The right column shows the sidechain motions that accompany these backbone motions, including motions of Arg664 toward ATP, as described in the text.

The resultant MFEP demonstrates that the activation loop backbone structure changes in four steps (Fig 1). In step (1), residues 660 through 663 move closer to the αC helix and the kinase’s N-lobe. Concurrently, the αC helix moves closer to the C-lobe. This apparently facilitates the formation of hydrogen bonds between the Nζ atom of Lys526 in the αC helix and the hydroxyl groups of Thr660 and Thr661. In step (2), the Ile654 side chain moves away from Arg649, clearing space for the side chain of pTyr657. This motion is accompanied by the sliding of the pTyr656 and pTyr657 backbone along the loop connecting the αEF and αF helices. In step (3), the pTyr656-pTyr657 backbone rotates to form the short antiparallel β-hairpin with Val679 and Tyr680 seen in the active crystal structure. This rotation accommodates two important sidechain motions, namely the inward migration of pTyr657 and Lys659, which allow for the formation of the network of hydrogen bonds in the active structure of FGFR2 kinase. Finally, in step (4), residues 660 through 663 move outward.

**Fig 2:**
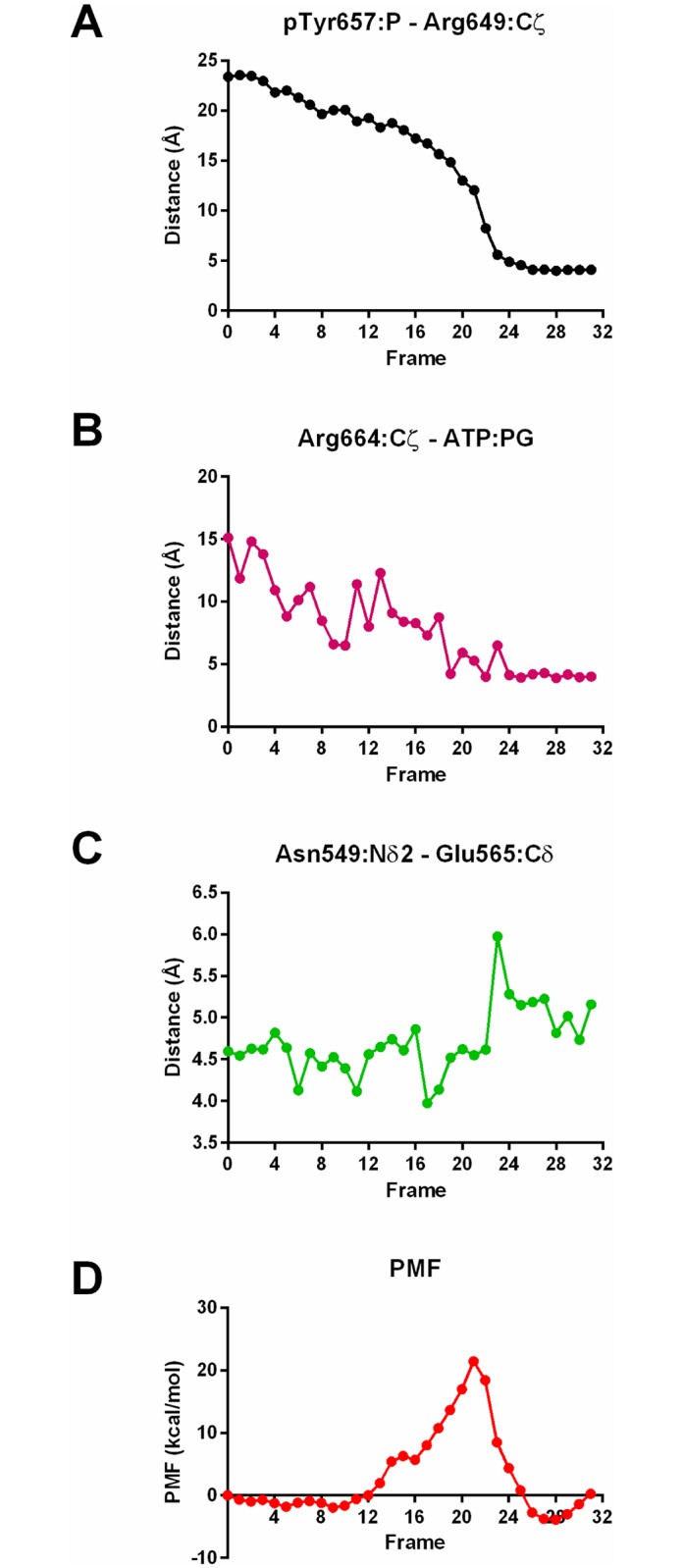
Key distances and potential of mean force along minimum free energy path. (A, B, C) Distances between key atoms over the course of the string method pathway. (D) Potential of mean force as a function of collective variables used in the string method study.

The major sidechain motion involved in the activation pathway is the inward motion of pTyr657 to make contact with Arg649, Arg625 and Lys659. However, we observed another important sidechain motion that occurs during the activation process, namely the motion of Arg664 toward ATP (Fig 2A). In the inactive conformation, and in the first 19 frames of the activation process, Arg664 points outward or makes contact with Glu527, enabled by proximity of the αC helix to the activation loop facilitated by the backbone motion of step (1). In the active conformation, however, Arg664 makes contact with the γ-phosphate of ATP stabilizing its position. We observed that the simulated motion of Arg664 toward ATP is synchronous with the motion of pTyr657 toward Arg649 (Fig 2B). Additionally, the dissolution of the hydrogen bond between Asn549 and Glu665, part of the regulatory “molecular brake” thought to prevent autoactivation of the kinase [26], occurs one frame after motion of pTyr657 (Fig 2C). This suggests that these two conformational changes might be structurally related as well, although a structural mechanism for this coupling is not readily apparent from this simulation study. In order to test our results, we performed the same algorithm but with an alternate set of CVs based on interatomic distances, discussed further in S1 Text.

We calculated the free energy as a function of the collective variables chosen in this string method study [34]. The plot of the potential of mean force (PMF) along the activation pathway indicates that there are two free energy wells corresponding to the inactive and active conformations (Fig 2D). The activation barrier occurs at frame 21, the same frame during which pTyr657 rotates inward and Arg664 approaches ATP, confirming that these two structural changes define the inactive and active states.

### Generating ensembles of catalytically inactive and catalytically active FGFR2

**Fig 3:**
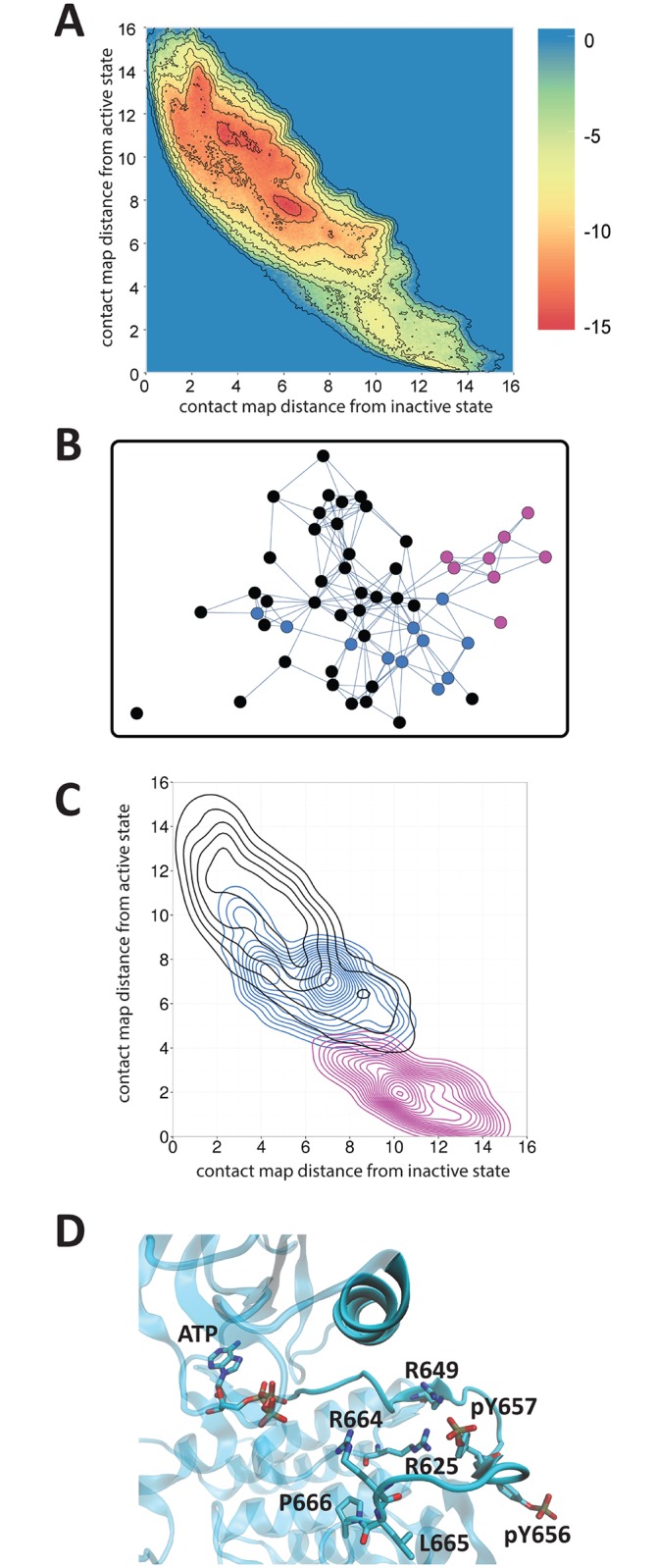
Results of metadynamics simulations. (A) Free energy landscape of metadynamics simulation. The brown dotted outline corresponds to the outermost blue contour in (C). (B) Network diagram of clusters from metadynamics simulation. Each cluster represents conformations whose backbone is within a 3.0 Å RMSD, and connected clusters are whose backbones are within 3.8 Å RMSD. Purple clusters represent those in which pTyr657 faces inward toward Arg649 and Arg625. In all these clusters, Arg664 faces into the active site (more than 50% of the conformations in that cluster feature Arg664:Cζ within 8 Å of ATP:Pγ), while Leu665 and Pro666 face outward without blocking the active site (the average distance between the center of mass of the side chain carbon atoms and the Cα atom of the catalytic base Asp626 is greater than 8 Å). Blue clusters represent those in which Arg664 faces inward and Leu665 and Pro666 face outward, even though pTyr657 does not face inward. All other clusters are black. (C) Contour map indicating the density of conformations in the black, blue and purple clusters, projected onto the same axes as in (A). Each contour represents an increase in density of 0.005. (D) Representative conformation in cluster 15, the most highly populated cluster in which pTyr657 faces inward.

In order to pinpoint general features of inactive and active conformations of the activation loop, without reference to a particular pathway, we ran a metadynamics simulation [36]. This generated a large pool of conformations similar to the inactive and active crystal structures as well as intermediate or related conformations. This used two contact map collective variables as the basis of the metadynamics simulations. Essentially, each collective variable corresponds to the number of interatomic contacts in the activation loop that are similar to contacts in the inactive or active structures, respectively; more details are discussed in Methods. The metadynamics simulation trajectories confirm the presence of two large free energy wells, roughly corresponding to inactive and active conformations of the protein (Fig 3A). The simulation was run for a long enough time to generate a large number of stable conformations with significantly divergent activation loop structures (see S1 Fig), rather than until convergence of the free energy landscape, which would likely have required unrealistic amounts of simulation time.

Clustering of the resulting pool of conformations based on a hierarchical agglomerative clustering scheme produced a final set of clusters in which no two conformations in any cluster were more than 3.0 Å apart, measured by RMSD of the activation loop backbone Cα atoms. This resulted in a total of 56 clusters. We then connected clusters whose conformations were no more than 3.8 Å apart (Fig 3B). We observed that eight of the 56 clusters represented “active” conformations, in which pTyr657 was rotated inward and made contact with Arg649, Arg625 and Lys659. In each of these clusters, two features were notable at the kinase’s active site (Fig 3D). First, the sidechains of Leu665 and Pro666 were rotated away from the active site. It is reasonable to conclude that this orientation of these side chains is necessary to allow catalysis because it enables the substrate tyrosine to insert near ATP and the presumed catalytic base, Asp626. A similar observation was made with regard to FGFR1, for which rearrangement of the activation loop prevents Arg661 and Pro663 from blocking the active site [11]. Second, in all eight of these clusters, Arg664 was pointed inward and made contact with the γ-phosphate of ATP, confirming that formation of this contact links with the inward motion of pTyr657.

An additional 11 out of 56 clusters featured both of these structural changes at the active site, namely Arg664 pointing into the active site and Leu665 and Pro666 pointing out of the active site. In these clusters, however, pTyr657 does not point inward to make contact with Arg649. Despite this, the backbone conformations of these clusters strongly resemble those of the eight clusters in which pTyr657 points inward. The average graph distance between each of these 11 clusters (active site ready, pTyr657-out) and the nearest pTyr657-in cluster is 2.4 Å, compared to 2.9 Å for all pTyr657-out clusters. This suggests that the backbone conformation common to both groups of clusters enables both Arg664 to point inward, and Leu665 and Pro666 to point outward from the ATP site. In turn, inward rotation of pTyr657, and the subsequent formation of contacts between pTyr657 and Arg649, Arg625, and Lys659, stabilizes this backbone conformation in order to preserve the catalytically permissive conformation of the activation loop near the active site. We examined the collective variable values for conformations in each of the three groups of clusters—active conformations, conformations with the pre-catalytic active site and the active-like backbone, and inactive conformations (Fig 3C). Notably, conformations with the active-like backbone form one of the smaller free energy wells (inside the brown dotted outline in Fig 3A) comprising the large free energy well corresponding to the inactive state. This free energy well is adjacent in CV space to the free energy well corresponding to the active state, suggesting that the active-like backbone structures are in an intermediate state between the fully inactive state and the active state.

**Fig 4:**
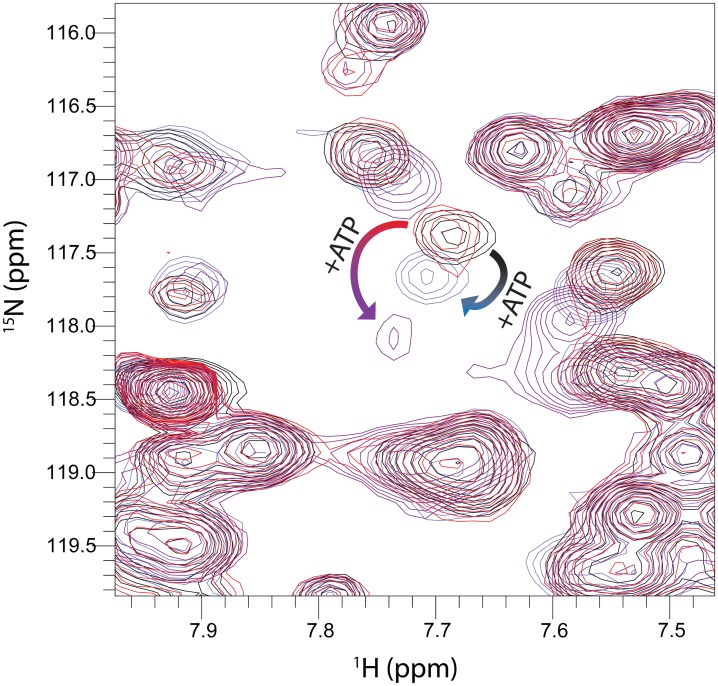
Region of ^15^N HSQC of FGFR2 kinase. Curved arrows indicate chemical shift perturbations that occur to the Arg664 shift as ATP analog is added. Black/blue spectra are of the unphosphorylated kinase, while red/purple spectra are of the phosphorylated kinase.

The phosphorylation of pTyr657 in the activation loop shifts the conformational dynamics of the loop to favor motion of Arg664 toward ATP in this simulation. We performed NMR experiments to examine the effect of activation phosphorylation on loop dynamics, by monitoring the chemical shift of the Arg664 HSQC cross-peak (Fig 4). When ATP was added to the unphosphorylated FGFR2 kinase, a chemical shift perturbation was seen for the Arg664 peak, indicating that the presence of ATP altered the chemical environment of Arg664. We attribute this to the Arg664 residue in fast exchange between the outward-pointing conformation and the inward-pointing conformation. As indicated by our simulations, the presence of ATP changes the distribution through electrostatic interactions with the Arg664 side chain. However, when ATP was added to the phosphorylated FGFR2 kinase, a larger chemical shift perturbation was seen for the Arg664 peak. HSQC peaks for R664 in spectra of the *apo* kinases (ATP-free phosphorylated and unphosphorylated) are similar, and the observed chemical shift perturbations in the ATP-bound samples lie along a straight line. These shift perturbations reflect change in the population between two endpoints [15]. The greater magnitude of the perturbation for the phosphorylated kinase is consistent with the simulation, with phosphorylation of the activation loop altering loop dynamics to enable greater interaction between the Arg664 guanidinium moiety and ATP.

### The role of Arg664 in FGFR2 kinase activity

**Fig 5:**
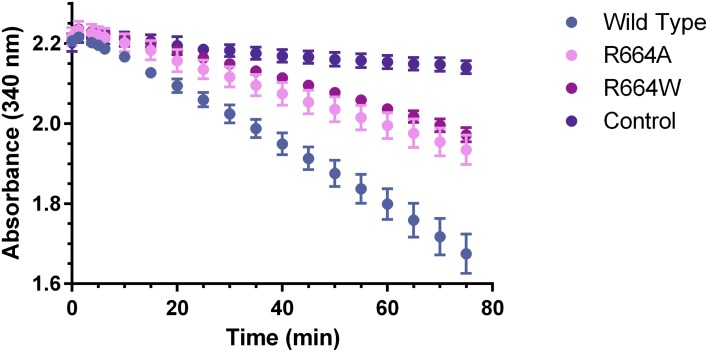
Kinetic assays of FGFR2 kinase. Kinetic assays were performed for wild-type FGFR2 kinase as well as R664A and R664W mutants. The control assay was performed without the presence of protein.

Conformations featuring an inward-pointing pTyr657 also show Arg664 in the active site in simulation, raising the likelihood that Arg664 is involved in mediating FGFR2 kinase activity. To test this, we generated two mutants of FGFR2 kinase, R664A and R664W. Kinetic assays were performed on the wild-type kinase and on each of these mutants (Fig 5). These assays showed that mutation of Arg664 to alanine or tryptophan caused a 52% and 60% decrease in autophosphorylation activity, respectively. Neither mutation has been demonstrated to definitively cause any pathology, but the R664W mutation has been found in a human colorectal tumor specimen [37], and bioinformatics analysis suggests that the mutation is highly deleterious to the protein’s function [37, 38]. Both mutations abolish the interaction between Arg664 and ATP, thus apparently reducing with the kinase’s catalytic activity.

**Fig 6:**
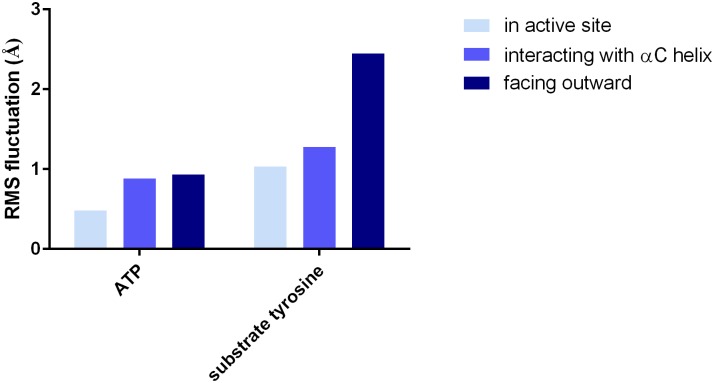
The effect of Arg664 on the precatalytic structure. Root-mean-square fluctuations of ATP and substrate tyrosine in simulations in which Arg664 was kept in the active site, away from the active site and pointing outward, or interacting with the αC helix.

The remaining unanswered question is precisely what role Arg664 plays in FGFR2 kinase catalytic activity. A previous crystallographic study of an FGFR3 mutant [24] also showed the homologous residue Arg655 in a similar position near the kinase’s active site, interacting with the γ-phosphate. The authors of that study proposed that the arginine residue stabilizes the position of the substrate tyrosine residue via π-cation interactions. To investigate this possibility in FGFR2 kinase, we performed MD simulations of FGFR2 kinase with a substrate peptide bound at the active site. Two simulations were performed, one simulation in which Arg664 was kept in the active site using a one-sided harmonic restraint, and another in which Arg664 was kept out of the active site using a one-sided harmonic restraint. The simulations showed that the positioning of the substrate tyrosine was indeed stabilized by the presence of Arg664 (S1 Movie). The tyrosine residue is neatly sandwiched by two arginine residues, Arg664 and Arg630. We observed that the root-mean-square fluctuations of the substrate tyrosine as well as of the ATP residue are higher for the simulation in which Arg664 was outside the active site (Fig 6). We also performed a third simulation with a restraint confining Arg664 to interact with Asp530, thus being close to the active site but not fully inside, as seen in several crystal structures [15, 26, 27, 39]. This simulation demonstrated an intermediate degree of substrate tyrosine confinement, though the ATP thermal motion was similar to that seen in the Arg664-out simulation (Fig 6).

**Fig 7:**
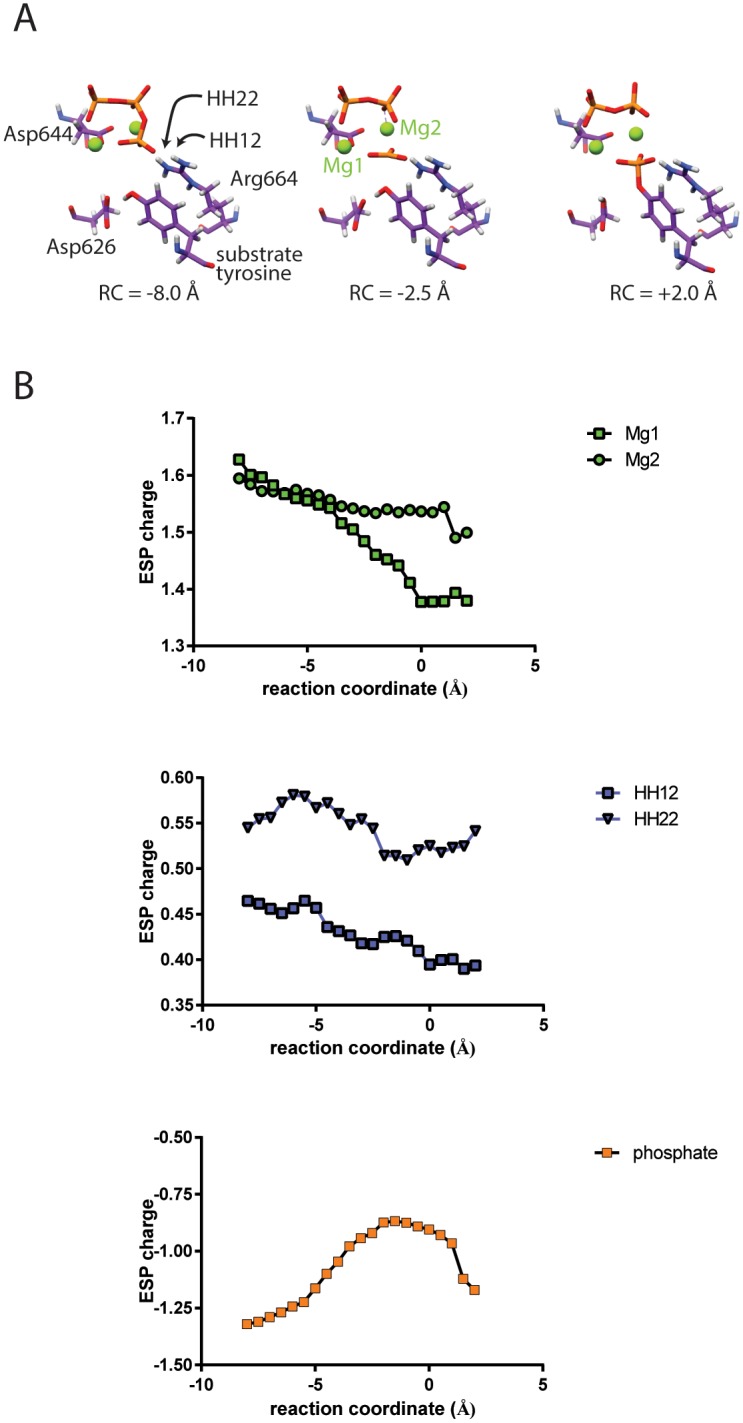
QM/MM studies of the FGFR2 kinase phosphotransfer reaction. (A) Three structures along the phosphotransfer reaction, with their corresponding reaction coordinate values. These structures correspond roughly to a reactant, transition state, and product state. (B) Electrostatic potential-derived partial charges of the phosphate group, two Mg^2+^ ions, and Arg664 guanidinium protons facing the phosphate group, as a function of the reaction coordinate.

To investigate whether Arg664 plays a role in the phosphotransfer reaction, we performed a series of QM/MM calculations in which the system transitioned from the reactant to the product state. At each step, the ESP-derived partial charges of the QM region atoms were calculated. The charges of the migrating phosphate group, the two Mg^2+^ ions, and the atoms of the guanidinium moiety are shown in Fig 7. As the phosphate group migrates toward the substrate tyrosine, its total charge and the charges of each individual phosphate oxygen become more positive, while the charges of the Mg^2+^ ions and protons of the guanidinium moiety of Arg664 become more negative. This is in accordance with our hypothesis that Arg664, like Mg^2+^, enables the progress of the phosphotransfer reaction by stabilizing the electron density of the phosphate group. More extensive QM/MM calculations are still needed to further explore the role of Arg664 on the phosphotransfer reaction.

## Discussion

Our work demonstrates that phosphorylation of the activation loop tyrosine residues alters FGFR2 kinase dynamics, allowing both the entry of the substrate tyrosine, and repositioning of Arg664 in the active site, and leading to facilitation of the catalytic reaction. Although there are numerous crystal structures of FGFR2 kinase, it has not been possible to infer mechanistic detail thus far because the static structures captured by X-ray crystallography do not encompass dynamic motions of the activation loop. For example, in one structure, the Arg664 side chain was not fully resolved (PDB 3CLY) [25], while in others its position appears to be influenced by the presence of ammonium sulfate (PDB 2PZP [26] and 1GJO [39]), and/or seen to form contacts with Asp530 (PDB 4J95, 4J96, 4J97, 4J98, 2PVY, 2PWL, 2PY3, 2PZ5, 2PZP, 2PZR, 2Q0B, 3B2T, 1OEC) [15, 26, 27, 39], as we observed in intermediate structures in the string method trajectory. The position of Arg664 seen in our simulations was observed previously in a crystal structure of the highly homologous FGFR3 kinase (PDB 4k33) [24], but not previously in FGFR2 kinase. Quite recently, after our simulations were completed, a crystal structure of FGFR2 kinase in complex with PLC-γ has been published showing Arg664 in contact with ATP and Leu665 and Pro666 facing outward [40]. Using string method and metadynamics simulations, we have found that this conformation is stabilized specifically by phosphorylation of the tyrosine residues in the activation loop. Moreover, MD simulations and kinase activity assays illustrate the functional role of Arg664 in kinase activity. Our results illustrate the need to complement these valuable crystal structures with dynamic information gleaned from simulation studies.

The pathway generated by the string method algorithm may also reveal several important features of FGFR2 kinase activation. Notably, the first step in the MFEP is the motion of residues 660 to 663 and the αC helix toward one another. This proximity enables hydrogen bonds to form between Lys526 and the hydroxyl and backbone carbonyl groups of Thr660 and Thr661. The importance of this motion in the pathway and its resultant proximity between the activation loop and the αC helix may contribute to the stable positioning of the substrate tyrosine. The role of the Lys526 residue has been established in studies demonstrating the significant gain of function caused by the K526E mutation responsible for Crouzon syndrome [26]. In this mutant, the Glu526 residue would be unable to form hydrogen bonds with Thr660 and Thr661. However, the K526E mutant can enhance catalytic activity through a similar mechanism to the wild-type, by formation of hydrogen bonds between the αC helix and activation loop. Glu526 can form hydrogen bonds with Arg664, reminiscent of contacts in the wild-type structure between Arg664 and Asp530. As our simulations indicate, the presence of Arg664 near the αC helix contributes to stability of the substrate tyrosine’s position, so the K526E mutant is likely to support this activating mechanism as well. Previous studies indicated that the K526E mutant significantly increases catalytic activity in both the unphosphorylated and phosphorylated state [26]. Since we hypothesize that in the phosphorylated state, Arg664 favors interaction with ATP, we propose that the K526E mutant favors Arg664 interacting with Glu526 predominantly when the phosphotyrosine residues are pointed outward, not interacting with Arg649 and Arg625. Since previous studies [15] and the current simulations indicate that the tyrosine-out state is predominant, even in the phosphorylated state, it is reasonable that the K526E mutation will significantly increase catalytic activity regardless of whether the kinase is phosphorylated.

The major limitation of the string method algorithm in this system is that the activation loop visits a large range of conformations, which are not all on the MFEP. Our metadynamics simulation explored a wide range of conformations, including some that diverged significantly from crystallographically observed structures. In particular, 13 of the 56 clusters contained structures in which pTyr656, not pTyr657, was pointed inward, making contact with Arg649 and Arg625 (S2 Fig). This conformation is of particular interest because previous studies in FGFR1 kinase have shown that Tyr653 (homologous to Tyr656 in FGFR2) is phosphorylated before Tyr654 (homologous to Tyr657 in FGFR2) [41]. Additionally, the monophosphorylated kinase, in which only Tyr653 is phosphorylated, has only a 50–100 fold increase in catalytic activity for *trans*-autophosphorylation compared to a 500–1000 fold increase in catalytic activity in the bisphosphorylated kinase relative to the unphosphorylated kinase [41]. Thus, understanding the structure of this monophosphorylated kinase might hold the key to designing inhibitors that selectively bind to this structure, which exists for some time before the bisphosphorylated kinase, is generated. While there are no experimentally-derived structures of a monophosphorylated kinase, our simulation-derived ensemble of pTyr656-inward structures which resemble the active conformation of the monophosphorylated kinase provides a foundation for exploring the unique features of these conformations of intermediate catalytic activity. Surprisingly, the conformations from the ensemble either did not feature Arg664 in the active site or had Leu665 and Pro666 blocking the active site. Further studies are needed to examine the structural features of the monophosphorylated kinase that enable catalytic activity.

The activation loop represents a formidable challenge in understanding RTK structure and dynamics, as it adopts not one structure but a large range of conformational states. As a result, many of the current experimental techniques are unable to probe completely the conformational space accessible to the activation loop. Within the range of crystal structures of FGFR2 kinase, the activation loop adopts many different conformations (S1 Fig). FGFR2 kinase is an especially useful system for investigating activation loop dynamics because other domains do not move significantly upon activation. Whereas in many RTKs, the αC helix undergoes movement upon activation, emphasizing its role in creating the catalytically active conformation of the active site, the αC helix in FGFR2 kinase does not move independently of the N lobe. Moreover, the N lobe structural change illustrated in crystal structures is itself subtle. We did not observe in our simulations any definite increase in proximity between the lobes in the active conformations compared to the inactive conformations. This suggests that interpreting the subtle differences between the inactive and active crystal structures warrants considerable caution. Because global conformational motions of the kinase lobes or secondary structures are not apparent, any determinants of catalytic activity are likely to be concentrated in the activation loop, making it a good choice for understanding how activation loop dynamics lead to kinase activation. Elucidating the structural mechanism by which phosphorylation of the activation loop enables catalytic activity is an important step toward designing specific inhibitors of FGFR2 kinase and other RTKs. Our work offers a model for activation loop dynamics, supported by experimental data, which may prove useful in better analyzing the dynamic changes that activate RTKs.

## Methods

### String method in collective variables

In order to generate input structures for the string method, we used the crystal structures of the inactive and active conformations of the FGFR2 kinase domain (PDB entries 2PSQ and 2PVF [26], respectively), superimposing them so as to minimize the RMSD between the structures. We used UCSF Chimera [42] to add missing terminal residues in each structure so that the final structures included residues 458 through 768 (seen in Fig 1). We used MODELLER [43] to add missing, low electron-density non-terminal loops, generating five structures for each missing loop and choosing the structure with the lowest score. The carbon atom of AMP-PCP in the catalytically active structure was changed to oxygen so that the bound ligand was ATP. Since the crystal structure of the catalytically inactive kinase does not include ATP, it was added to the structure based on its position in the active kinase. All crystallographic water molecules and other precipitant molecules in the crystal structures were removed, as was the peptide substrate from the structure of the active kinase. The LEAP program [44] was used to generate the remainder of each structure. LEAP added phosphate groups to the tyrosine residues of the activation loop and the kinase hinge. Parameters for the phosphotyrosine residue and ATP were based on [45] and [46] respectively. In each of these structures, residue 491, which had been mutated in the crystal structure from Cys to Ala, is reverted to Cys. The correct number of Na^+^ counter ions were added to each structure, as well as enough TIP3P solvent to create a 10 Å buffer between the protein edge and the box wall. For all simulations, the AMBER99SB force field [47] was used. Hydrogen bonds were constrained using the SHAKE algorithm [48]. Long-range electrostatics were computed using the particle mesh Ewald algorithm [49]. All molecular dynamics simulations used a 2 fs time step.

The simulation boxes containing the inactive and active conformations were each minimized using NAMD [50] for 1000 steps of conjugate gradient minimization, keeping 500 kcal mol^-1^Å^-2^ restraints on the CA atoms of the protein, followed by 2500 steps of minimization without restraints. The initial path for the string method in collective variables is derived from the zero-temperature string (ZTS) method [51, 52], which produces the minimum energy path of the conformational transition between the inactive and active structures. It has been shown that the minimum energy path is a good choice for input to the string method in collective variables, as the minimum energy path (MEP) produced by the ZTS method is likely to be similar to the minimum free energy path (MFEP) produced by the string method in collective variables [35]. Before implementing the ZTS method, the water molecules from the inactive and active structures are removed. A pathway of four structures is created by inserting two linearly interpolated structures between the inactive and active structures. These four structures then undergo 100 iterations of the ZTS method. In each iteration, each structure is minimized in AMBER [44] with 20 steps of steepest descent minimization, using an infinite cutoff for short-range interactions, followed by reparametrization of the string of structures so that they are equally spaced from one another along the string in conformational space. After 100 iterations, the number of structures in the string is doubled by interpolating one structure between each pair of successive structures, and two structures between the middle pair of structures in the string, and the procedure is repeated. This continues until the ZTS method runs for a string of 256 structures. Thirty-two equally spaced structures are extracted from this path (starting with image 7 and ending with image 255, the last image in the path) for input into the string method in collective variables.

LEAP is run on each of these 32 structures to add back Na^+^ counter ions and water molecules as before, followed by rotation and translation of the box to align the protein molecules in each box to one another. Each structure is then minimized in NAMD for 2000 steps with 10 kcal mol^-1^Å^-2^ restraints on the protein atoms, followed by gradual heating to 300 K over 600 ps with the same restraints using a Langevin thermostat with a damping coefficient of 1 ps^-1^. Each structure is equilibrated in the NPT ensemble for 2 ns using a Berendsen barostat with a target of 1 bar and a compressibility of 4.57 × 10^−5^ bar^-1^. The resulting structures are used as input for the string method algorithm.

The collective variables (CVs) for the string method include the Cα atoms of the αC-helix (residues 526–541) and the activation loop (residues 644–683), as well as sidechain atoms of residues postulated to be important in the mechanism of activation (two sidechain atoms of the “molecular brake” [26], Asn549:Nδ2 and Glu565:Cδ; the phosphorus atoms of the phosphotyrosine residues of the activation loop; the Cζ atom of Arg649 in the activation loop which makes contact with pTyr657 in crystal structures of the active conformation; and the Nζ atoms of Lys658 and Lys659 which make contact with pTyr656 and pTyr657). During the simulations, these atoms are constrained to target values (denoted *z*_1_*, *z*_2_*, …, *z*_n_* for each of the *n* collective variables) with a 1.0 kcal mol^-1^Å^-2^ restraint, with the initial target values extracted from the equilibrated structures. MD simulations are run for each image in the string independently. After every 10 steps, the target values *z*_i_* are evolved according to the equation

$$z_{i}^{*}(t+dt)=z_{i}^{*}(t)-\gamma^{-1}m^{-1}\frac{\partial F}{\partial z_{i}}dt$$ (1)

where *γ* is a friction coefficient given by 125 ps^-1^, *m* is the mass (taken for simplicity to be identical to the mass of a carbon atom) and *F* is the free energy. The derivative of free energy with respect to a given CV is approximated by the average value

$$\frac{\partial F}{\partial z_{i}}=k\left(\langle z_{i}^{*}(t)-z_{i}\rangle \right)$$ (2)

where *z*_*i*_ is the instantaneous value of the CV and <…> denotes the ensemble average over 10 steps. The target values are thus updated for each CV for each image, which together form a string, a path in CV space. After the update step, the string is reparameterized such that the new target values are still on the same string in CV space, but equidistant from one another; this is easily performed by calculating the optimal distance between target values for each image and changing the target values, as noted in [35]. This process is continued until the set of target values does not change significantly over time (represented by asymptotic behavior of the RMSD of target values from their initial values; see S3 Fig), suggesting that the string now represents a minimum free energy path in collective variable space. Throughout the simulations, we prevented translation and rotation of the protein by adding 0.5 kcal mol^-1^Å^-2^ restraints on the backbone atoms of the protein except those in the αC-helix or the activation loop.

In order to visualize the final pathway, we ran simulations with 20 kcal mol^-1^ Å^-2^ constraints on the restrained atoms to guide them toward the final target values. The PMF is calculated by running simulations with 1 kcal mol^-1^ Å^-2^ constraints on the restrained atoms at the final values from the string method algorithm. This allows calculation of ∂*F*/∂*z*_*i*_ for each CV, then enabling the generation of a PMF curve whose equation is given by

$$F(\alpha )=\int_{0}^{\alpha }\underset{i}{\sum }\frac{\partial F}{\partial z_{i}}\frac{\partial z_{i}}{\partial \alpha }d\alpha$$ (3)

We calculate the value of ∂*z*_*i*_/∂*α* using centered differences (or forward or backward differences for the starting and ending frames, respectively), and we calculate the integral using the trapezoidal method.

A second run of the string method in collective variables was run with a different set of CVs. For this second run, the CVs were interatomic distances between the centers of mass of residues in the αC-helix and activation loop, as well as sidechain atoms of the “molecular brake.” The overall conclusions from this run were similar and are summarized in the supporting information, and S1 Table, and S4 and S5 Figs.

### Metadynamics in contact map space

The starting and ending target values of the CVs pertaining to the activation loop were used to run metadynamics simulations [36] using distances in contact map space from the active and inactive states as the CVs. We calculated interatomic distances between all atoms in the activation loop whose coordinates were CVs in the string method simulations. We then selected the subset of those distances which changed between the inactive state (frame 0) and the active state (frame 31) from being less than 8 Å to greater than 8 Å, or vice versa, and in which the greater distance was at least 1.5 times larger than the smaller distance. This subset, containing 31 interatomic distances, was used to define the contact map. Then we used distances in contact map space as the collective variables in a metadynamics simulation. For a given conformation,

$$d={\left(\underset{r}{\sum }\left(\frac{1-{\left(\left(r-d_{0}\right)/r_{0}\right)}^{6}}{1-{\left(\left(r-d_{0}\right)/r_{0}\right)}^{12}}\right)\right)}^{1/2}$$ (4)

where *r* is one of the 31 interatomic distances, *r*_0_ = 8 Å, and *d*_0_ is the reference contact distance in the inactive or active state.

As the metadynamics simulation progressed, every 500 steps, a 2D Gaussian hill with height 0.7 kcal mol^-1^ and width 0.1 was added, centered at the current value of the contact map distance from the active and inactive conformations, respectively. We used well-tempered metadynamics [53], with a bias temperature of 4200 K, which determined the height of the Gaussian at each step. A grid with spacing 0.002 was used to store the Gaussian hills. Additionally, the metadynamics simulation was significantly accelerated by using 10 simultaneous walkers [54] which shared a collective set of Gaussian biases. The total simulation time for all walkers exceeded 3 μs. The metadynamics simulation was performed using NAMD 2.9 [50] with PLUMED 2.1 [55].

All calculations were performed on local workstations as well as TACC Stampede and Maverick [56].

### DNA cloning and protein expression

Constructs of FGFR2 kinase for NMR and kinetics studies were based on [15]. Human full-length FGFR2 cDNA was purchased from Sino Biological Inc. (Beijing, China), and the kinase domain fragment was extracted by restriction digest with EcoRI and XhoI, generating residues 458 through 768, which were subsequently cloned into the pRSFDuet vector. Subsequent mutations were introduced into the construct via site-directed mutagenesis using a QuikChange II XL kit, and sequences were confirmed by DNA sequencing. All constructs used in this study included a C491A mutation that aided in protein expression [15].

Protein expression was carried out in BL21-DE3 RIPL cells. Cells were grown either in Terrific Broth (Sigma-Aldrich) or in M9 minimal media supplemented with ^15^N NH_4_Cl for NMR studies, and were induced at OD 0.6–0.8 with 1 mM IPTG overnight at 20°C. Cells were lysed and protein was purified using TALON metal affinity resin (Clontech). To generate non-phosphorylated sample, trace phosphorylation was removed by alkaline phosphatase (FastAP, Thermo Scientific), followed by purification by size-exclusion chromatography. To prepare phosphorylated sample, 10 mM ATP and 5 mM MgCl_2_ were added to FGFR2 kinase and autophosphorylation was allowed to occur overnight at 4° followed by exhaustive dialysis to remove excess ATP.

### NMR studies

For NMR studies, we used constructs in which all tyrosine residues except Tyr657 in the activation loop, shown to be sufficient for full catalytic activity [15], were mutated to phenylalanine. Additionally, we used the A648T mutation to improve protein expression and stability for the NMR experiments. NMR HSQC spectra of FGFR2 kinase with the A648T mutation were obtained from a sample of 150 μM protein in a buffer consisting of 20 mM HEPES pH 7.4 and 150 mM KCl. Assignments for the A648T construct were transferred from published assignments [15]. The assignment of the HSQC cross peak corresponding to R664 was confirmed using selective un-labeling experiments by adding ^14^N-arginine to the growth media, as well as using selective labeling experiments by adding ^13^C-glycine and ^15^N NH_4_Cl to the growth media, generating a spectrum which contained cross peaks from residues immediately C-terminal to the glycine residues in an ^1^H-^15^N HNCO plane.

### Kinetic assays

In kinetic assays, the tyrosine residues of the kinase domain were kept intact, with C491A as the only mutation retained to allow for protein expression. Kinetic assays of autophosphorylation were performed by coupling the hydrolysis of ATP to the oxidization of NADH through enzymes in the glycolytic pathway, as discussed in [57]. The assay contained 500 nM of unphosphorylated FGFR2 kinase, along with 1 mM ATP, 20 mM MgCl_2_, 1 mM phosphoenolpyruvate, 45–70 units LDH, 30–50 units PK, and 416 μM NADH. The kinase activity was monitored by measuring absorbance at 340 nm, which reflects the amount of NADH in the sample that has not yet been oxidized.

### Simulations of substrate tyrosine positioning

The starting structure for MD simulations of the kinase with the bound substrate peptide was taken from a structure of the kinase performing autophosphorylation (PDB 3CLY [25]). After making copies of the unit cell using UCSF Chimera [42], a portion of the substrate-acting kinase is retained, with the sequence TTNEEYLDL, while the remainder of that copy of the kinase is discarded. The ACP molecule was changed to ATP, and missing non-terminal segments of the enzyme-acting kinase were built using MODELLER as described above. Missing atoms were added with LEAP, followed by addition of Na^+^ counter ions and solvent to generate a box with an 8 Å margin surrounding the protein on all sides. The box was minimized in AMBER with 500 steps of steepest descent minimization followed by 1500 steps of conjugate gradient minimization, all with 500 kcal mol^-1^Å^-2^ restraints on the protein. This was followed by minimization with restraints on only the Cα atoms, using 500 steps of steepest descent and 500 steps of conjugate gradient minimization. The box was heated in AMBER to 300 K over 20 ps, with 10 kcal mol^-1^ Å^-2^ restraints on the Cα atoms, and NPT equilibration was performed for 2 ns, with 5 kcal mol^-1^ Å^-2^ restraints on the atoms of the active site (including Asp626, Asp644, Arg664, the substrate tyrosine, ATP, the two Mg^2+^ ions, and five water molecules which were all within 5 Å of both Mg^2+^ ions). These simulations used the same parameters used for the simulations described above.

To generate starting configurations for simulations for comparison of tyrosine stabilization by Arg664 positioning, we ran preparatory simulations to move Arg664 toward a given position while the active site atoms were kept fixed with a 5 kcal mol^-1^ Å^-2^ restraint. To study the effect of Arg664 near the active site, Arg664 was moved toward ATP by placing a harmonic restraint on the Arg664:Cζ—ATP:Pγ distance, with an equilibrium value of 4.5 Å, whose force constant increased linearly over 200 ps from 0 to 10 kcal mol^-1^ Å^-2^, followed by another 300 ps of simulation with the restraint constant. During the subsequent production simulations in which the root-mean-square fluctuations of ATP and the substrate tyrosine were measured, a half-harmonic potential was placed, with a force constant of 5 kcal mol^-1^ Å^-2^, whenever the distance increased beyond 6 Å. To study the effect of having Arg664 far away from the active site, a second simulation was run to move Arg664 away, using a similar harmonic restraint as above but with an equilibrium value of 25 Å; the production runs included a half-harmonic restraint activated when the distance went below 10 Å. To study the effect of having Arg664 bound to Asp530, a preparatory simulation included a harmonic restraint on the Arg664:Cζ—Asp530:Cγ distance with an equilibrium value of 4.5 Å, followed by production runs with a half-harmonic potential activated if the distance increased beyond 6 Å.

### QM/MM studies

The starting structure for QM/MM studies was identical to that used in the substrate positioning simulation to study the effect of positioning Arg664 in the active site. The QM region included the three phosphate groups of ATP; two Mg^2+^ ions; the sidechains of Asp626, Asp644, Arg664 and the substrate tyrosine; and five water molecules that were within 5 Å of both Mg^2+^ ions. The rest of the system was treated at the MM level using the same AMBER force field. The QM region was studied using density functional theory with the B3LYP exchange correlation functional, using the cc-pVDZ basis set [58]. Link atoms were used to connect the two regions. All QM/MM calculations were performed using NWChem 6.3 [59]. First, the geometry was optimized using an alternating optimization scheme, in which the QM region undergoes 10 steps of optimization, followed by optimization of the MM solute region while the QM region is represented by ESP-derived charges, and then optimization of the solvent. This alternating optimization scheme continues until convergence. After optimization, we used the reaction coordinate driving method [60] to drive the system from the reactant state to the phosphorylated product state. We used the reaction coordinate RC = *d*_1_ − *d*_2_ − *d*_3_, where *d*_1_ is the ATP:Pβ–ATP:Pγ distance, *d*_2_ is the ATP:Pγ—Tyr:OH distance, and *d*_3_ is the Tyr:HH—Asp626:Oδ2 distance. The system was optimized at each step with harmonic restraints on the reaction coordinate that successively increased its value until the reaction completed.

## Supporting information

S1 Fig “Monophosphorylated” conformations. Representative structures from 12 clusters of metadynamics trajectory structures in which pTyr656 is pointed inward, and not pTyr657.
(EPS)

S2 Fig Conformational heterogeneity from metadynamics. Superimposed representative structures of 56 clusters of metadynamics trajectory structures.
(EPS)

S3 Fig Convergence of the string method. Root-mean-square displacement of string in CV space from its original position in CV space.
(PNG)

S4 Fig Convergence of the string method (alternate CVs). Root-mean-square displacement of string in CV space from its original position in CV space, for the set of CVs discussed in S1 Text.
(TIF)

S5 Fig Motion of pTyr657 and Arg664 in string method algorithm using alternate CVs. Distances between key atoms over the course of the string method pathway using alternate CVs discussed in S1 Text.
(TIF)

S1 Text String method algorithm using alternate CVs. Discussion of string method algorithm using an alternate set of CVs to confirm results discussed in main text.
(DOCX)

S1 Table Atom groups used for CVs in alternate string method algorithm. (DOCX)

S1 Movie Simulation of the precatalytic structure with Arg664. Simulation of the FGFR2 kinase precatalytic complex with Arg664 in the active site.
(MPG)

S2 Movie Simulation of the precatalytic structure without Arg664. Simulation of the FGFR2 kinase precatalytic complex with Arg664 constrained to remain outside of the active site.
(MPG)
