# Supplementary figures and images for: Effects of FGFR2 kinase activation loop dynamics on catalytic activity
Source: PLoS Comput Biol. 2017 Feb 2;13(2):e1005360. doi: 10.1371/journal.pcbi.1005360 (PMC5313233; doi:10.1371/journal.pcbi.1005360)

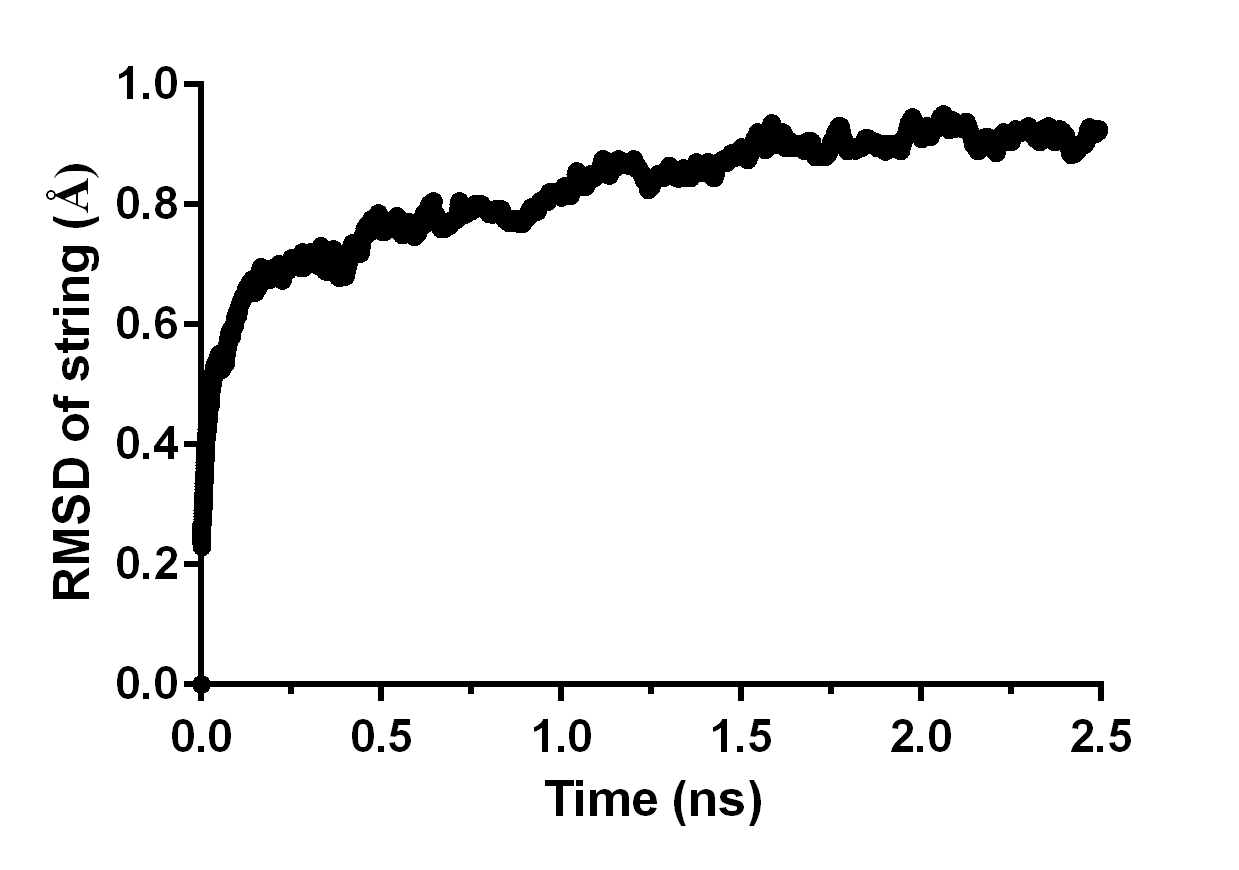

Supplement: S3 Fig — Root-mean-square displacement of string in CV space from its original position in CV space. (PNG) [file pcbi.1005360.s003.png]

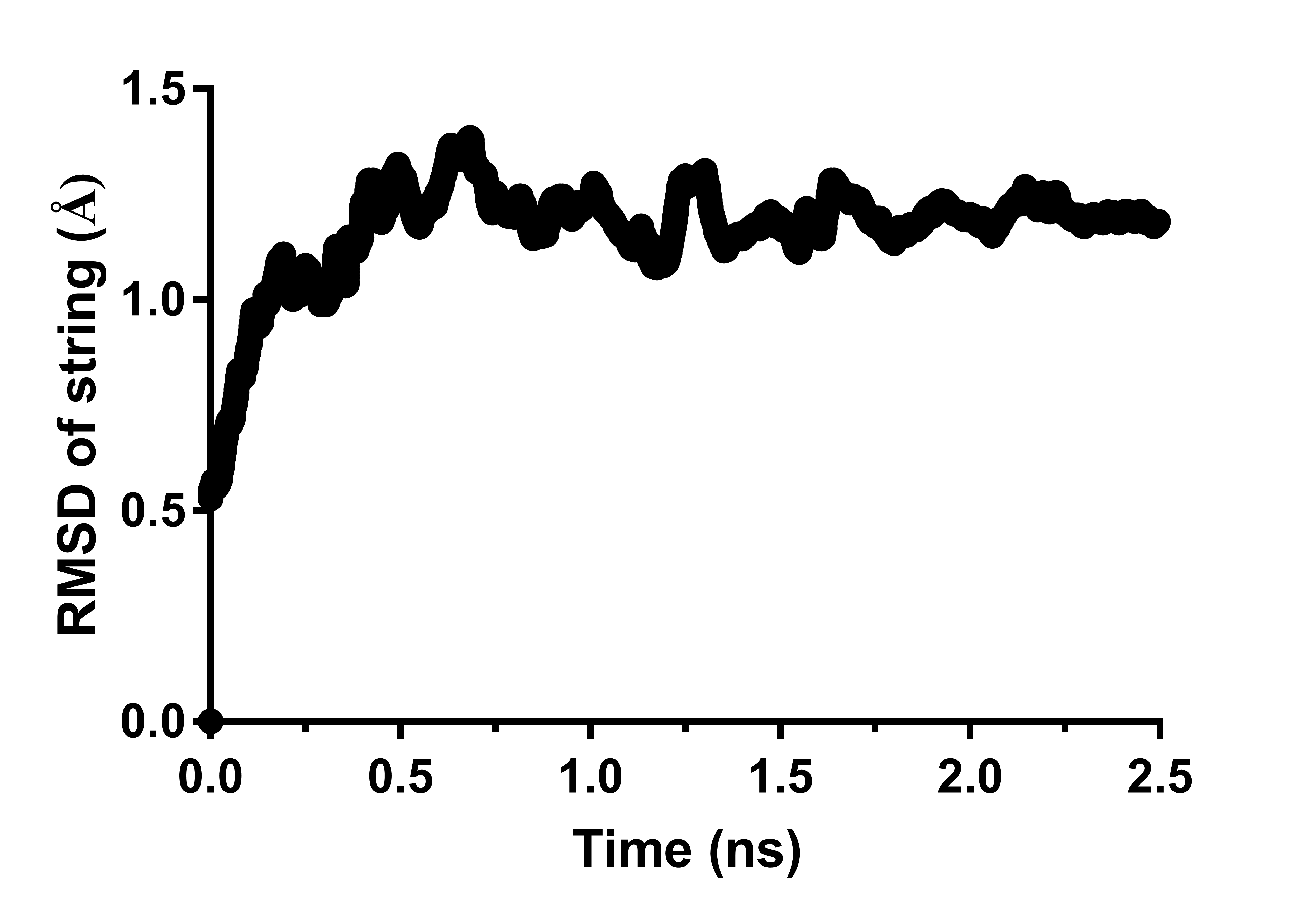

Supplement: S4 Fig — Root-mean-square displacement of string in CV space from its original position in CV space, for the set of CVs discussed in S1 Text. (TIF) [file pcbi.1005360.s004.tif]

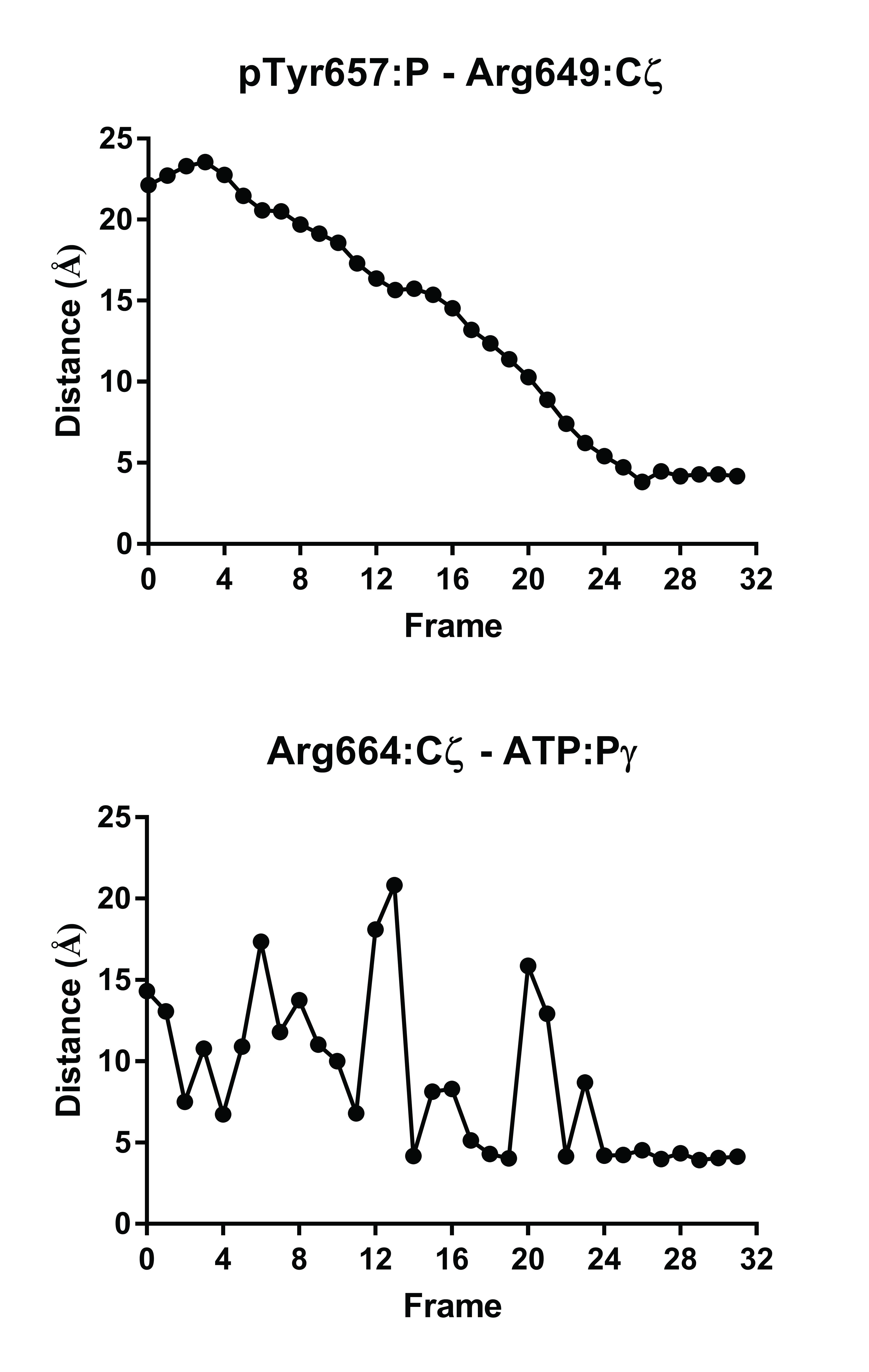

Supplement: S5 Fig — Distances between key atoms over the course of the string method pathway using alternate CVs discussed in S1 Text. (TIF) [file pcbi.1005360.s005.tif]
