# Supplementary material for: Effects of FGFR2 kinase activation loop dynamics on catalytic activity
Source: PLoS Comput Biol. 2017 Feb 2;13(2):e1005360. doi: 10.1371/journal.pcbi.1005360 (PMC5313233; doi:10.1371/journal.pcbi.1005360)
Supplement: S1 Text — Discussion of string method algorithm using an alternate set of CVs to confirm results discussed in main text. (DOCX) [file pcbi.1005360.s006.docx]

# Effects of FGFR2 Kinase Activation Loop Dynamics on Catalytic Activity: Supplementary Information – S1 text

Additional simulation with alternate collective variables.

A second run of the string method in collective variables was run with a different set of CVs. For this second run, the CVs were interatomic distances between the centers of mass of residues in the αC-helix and activation loop, as well as sidechain atoms of the “molecular brake.” A complete list of the atoms involved in the CVs are listed in S1 Table. The algorithm proceeds identically to that of the algorithm discussed in the main text, but the evolution step after every 10 steps proceeds by the equation

where $M_{ij}$ is the metric tensor, $x_{k}$ represents a Cartesian coordinate of one of the atoms in the simulation, and represents the ensemble average over 10 MD steps. The index $k$ runs from 1 to $3N$, where $N$ is the number of atoms in the simulation, though many of the partial derivatives are zero since most of the atoms in the simulation are not included in any CV. Since all the CVs are distances between the centers of mass of two groups of atoms, the formula for $M_{ij}$, if $i\neq j$ , is given by

$$M_{ij}=m^{-1}\frac{N_{\text{shared}}}{N_{i}N_{j}}\frac{\mathbf{r}_{i}\cdot\mathbf{r}_{j}}{z_{i}z_{j}}, i\neq j$$

where $N_{i}$ and $N_{j}$ are the number of atoms contributing to the center of mass of the intersecting atom groups that are part of CVs $i$ and $j$, $N_{shared}$ is the number of atoms in the intersection, $z_{i}$ and $z_{j}$ are the values of the CVs, and $\mathbf{r}_{i}$ and $\mathbf{r}_{j}$ are the vectors corresponding to the centers of mass of the two intersecting groups. The diagonal entries of the matrix $M_{ij}$ are given by

$$M_{ii}=\frac{1}{m_{1}N_{1}}+\frac{1}{m_{2}N_{2}}$$

where $N_{1}$ and $N_{2}$ are the number of atoms in each of the two atom groups used to calculate the CV $i$, and $m_{1}$ and $m_{2}$ are the masses of the atoms in each atom group. (Note that each atom group is comprised of atoms of identical mass, though some CVs are distances between two groups of atoms whose masses are different.) If CVs $i$ and $j$ share no atoms, then the value of $M_{ij}$ is zero.

This algorithm was run for 2.5 ns until the RMSD of the target CV values from their initial values converged (see S4 Fig). In order to visualize the final pathway, we ran simulations with 1 kcal mol^-1^ Å^-2^ constraints on the restrained distances to guide them toward the final target values.

The results with this alternate set of CVs shows a similar activation pathway to the results of the algorithm discussed in the main text. Because the CVs are interatomic distances rather than atomic positions, the pathway is less smooth than the path resulting from the algorithm using positions. However, the same pattern is evident: as pTyr657 approaches Arg649, Arg664 approaches the active site of the phosphotransfer reaction and makes contact with the γ-phosphate of ATP (S5 Fig).
