# Supplementary material for: Effects of FGFR2 kinase activation loop dynamics on catalytic activity
Source: PLoS Comput Biol. 2017 Feb 2;13(2):e1005360. doi: 10.1371/journal.pcbi.1005360 (PMC5313233; doi:10.1371/journal.pcbi.1005360)
Supplement: S1 Table — (DOCX) [file pcbi.1005360.s007.docx]

**S1 Table.** Atom groups used for CVs in alternate string method algorithm.

|  | **Group 1** | **Group 2** |
| --- | --- | --- |
| 1 | Asn549:Nδ2 | Glu565:Cδ |
| 2 | Asn549:Nδ2 | α carbons of αC helix residues (526-541) |
| 3 | Arg649:Cα | α carbons of αC helix residues (526-541) |
| 4 | pTyr657:P | Arg649:Cζ |
| 5 | pTyr657:P | Lys659:Nζ |
| 6 | pTyr656:P | Lys658:Nζ |
| 7 | α carbons of initial activation loop residues (644-650) | Ile651:Cα and Asn652:Cα |
| 8 | α carbons of initial activation loop residues (644-650) | Asn653:Cα and Ile654:Cα |
| 9 | α carbons of initial activation loop residues (644-650) | Asp655:Cα and pTyr656:Cα |
| 10 | α carbons of initial activation loop residues (644-650) | pTyr657:Cα and Lys658:Cα |
| 11 | α carbons of initial activation loop residues (644-650) | Lys659:Cα and Thr660:Cα |
| 12 | α carbons of initial activation loop residues (644-650) | Thr661:Cα and Asn662:Cα |
| 13 | α carbons of initial activation loop residues (644-650) | Gly663:Cα, Arg664:Cα and Leu665:Cα |
| 14 | α carbons of final activation loop residues (666-673) | Ile651:Cα and Asn652:Cα |
| 15 | α carbons of final activation loop residues (666-673) | Asn653:Cα and Ile654:Cα |
| 16 | α carbons of final activation loop residues (666-673) | Asp655:Cα and pTyr656:Cα |
| 17 | α carbons of final activation loop residues (666-673) | pTyr657:Cα and Lys658:Cα |
| 18 | α carbons of final activation loop residues (666-673) | Lys659:Cα and Thr660:Cα |
| 19 | α carbons of final activation loop residues (666-673) | Thr661:Cα and Asn662:Cα |
| 20 | α carbons of final activation loop residues (666-673) | Gly663:Cα, Arg664:Cα and Leu665:Cα |
